# Propionic Acidemia: Diagnosis and Neuroimaging Findings of This Neurometabolic Disorder

**Published:** 2014

**Authors:** Parvaneh KARIMZADEH, Narjes JAFARI, Farzad AHMAD ABADI, Sayena JABBEDARI, Mohammad-Mahdi TAGHDIRI, Mohammad-Reza ALAEE, Mohammad GHOFRANI, Seyed Hassan TONEKABONI, Habibeh NEJAD BIGLARI

**Affiliations:** 1 Pediatric Neurology Research Center, Shahid Beheshti University of Medical Sciences (SBMU), Tehran, Iran; 2 Pediatric Neurology Excellence Center, Pediatric Neurology Department, Mofid Children Hospital, Faculty of Medicine, Shahid Beheshti University of Medical Sciences (SBMU), Tehran, Iran; 3 Department of Pediatric Endocrinology, Faculty of Medicine, Shahid Beheshti University of Medical Sciences, Tehran, Iran

**Keywords:** Propionic academia, Neurometabolic disorder, Developmental delay, Early detection

## Abstract

**Objective:**

Propionic acidemia is one of the rare congenital neurometabolic disorders with autosomal recessive inheritance. This disorder is caused by a defect in the propionyl-CoA carboxylase enzyme and can be presented with life-threatening ketoacidosis, lethargy, failure to thrive, and developmental delay.

**Materials & Methods:**

The patients diagnosed as having propionic acidemia in Neurology Department of Mofid Children’s Hospital in Tehran, Iran, between 2002 and 2012 were include in our study. This disorder was confirmed by clinical manifestations, neuroimaging findings, and neurometabolic assessment in the reference laboratory in Germany. Our study was conducted to define the sex, age, gender, past medical history, developmental status, clinical findings, and neuroimaging manifestations in 10 patients with propionic acidemia.

**Results:**

Seventy percent of patients were offspring of consanguineous marriages. In this study, only one patient had microcephaly at birth, but at detection time, 30% of patients had head circumference and weight below the 3rd percentile.

The patients were followed for approximately 5 years and this follow-up showed that the patients with early diagnosis had a more favorable clinical response. Neuroimaging findings included brain atrophy, white matter and globus pallidus involvement.

**Conclusion:**

Finally we suggest that early diagnosis and treatment have an important role in the prevention of disease progression.

## Introduction

Propionic acidemia is a disorder of organic acid metabolism with recessively inheritance, characterized by a spectrum of clinical and biochemical manifestations. The main feature of this disorder is metabolic acidosis, which is useful in differentiating it from non-ketotic hyperglycinemia (1). 

Diagnosis of propionic acidemia is made by mass spectrometry, gas chromatography, and urine excretion of methylcitrate and 3-hydroxy-propionate (2).

The main features of this disorder are based on recurrent vomiting, ketosis, hypotonia, difficult feeding, lethargy, hyperglycinemia, episodes of seizure and hyperammonemia during the acute episodes of this disorder (3,4).

There are a broad spectrum of neurological symptoms and signs, including structural abnormalities, neurodevelopmental delay, metabolic stroke-like episodes, cranial nerve abnormalities, and seizures (5). When the metabolic demand is increased, patients’ deterioration occurs (6).

In this study, we present 5 years of experience about propionic acidemia in Pediatric Neurology Research Center of Mofid Children’s Hospital in Tehran, Iran and we describe 10 cases of this disorder with clinical symptoms and neuroimaging findings.

## Materials & Methods

Patients were diagnosed as propionic acidemia based on clinical manifestations, neuroimaging findings, and finally laboratory assessment of propionic acid at metabolic disorders laboratory in Germany. Assessment information was collected as age, gender, past medical history, development status, general appearance, and clinical and neuroimaging findings. The data were analyzed using descriptive method and no statistical testing was applied as observational study. 

Prevention included genetic counseling and reducing new cases of disorder.

## Results

Ten patients were included in this study. They were evaluated in terms of clinical, laboratory, and neuroimaging findings. Seven patients were male and three were female, and the age range was 1-48 months. The earliest case was diagnosed at birth and the late case was diagnosed at age of 4. The mean age of patients at detection time was 31.2 months. Seventy percent of the patients were offspring of consanguineous marriages. In 60% of patients, the family history of neurological disorder was positive (mental retardation and seizure). The first and chief complaint in 70% of the patients was neurological disorders, such as developmental delay and seizure. Two patients had cognitive regression and were unresponsive to sound. One patient had autistic manner and another had fear and hallucination. Thirty percent of patients had a history of neonatal hospitalization because of recurrent vomiting and narcosis. Thirty percent of patients had a history of neonatal hospitalization because of respiratory disorders. Growth index at birth in all patients was normal, except in one patient with microcephaly, but at detection time, 30% of patients had head circumference and weight below the 3rd percentile. 

Developmental delay and developmental regression were seen in 90% and 60% of the patients, respectively. Twenty percent of patients had a specific face (micrognathia and saddle nose). Thirty percent of patients had movement disorder as dystonia. Hypertonicity was seen in 30% of them, and 40% of patients with propionic aciduria had hypotonicity. 

Fifty percent of patients had seizure (40% had tonic seizure and one patient had GTC). None of the patients had refractory seizure. Thirty percent of patients had agitation and irritability. Fundoscopic examination was normal, and there was no certain point in other physical examination (hair and skin, face, chest, and abdomen). 

In lab data, 40% of patients had decreased levels of hemoglobin, 30% had elevated levels of AST and ALT, 60% had increased levels of ammoniac and lactate, 20% had decreased level of blood sugar, and 40% had metabolic acidosis. Other biochemistry tests were normal in all of the patients. In the sonography of one patient, multiple renal stones were reported. 

**Fig1:**
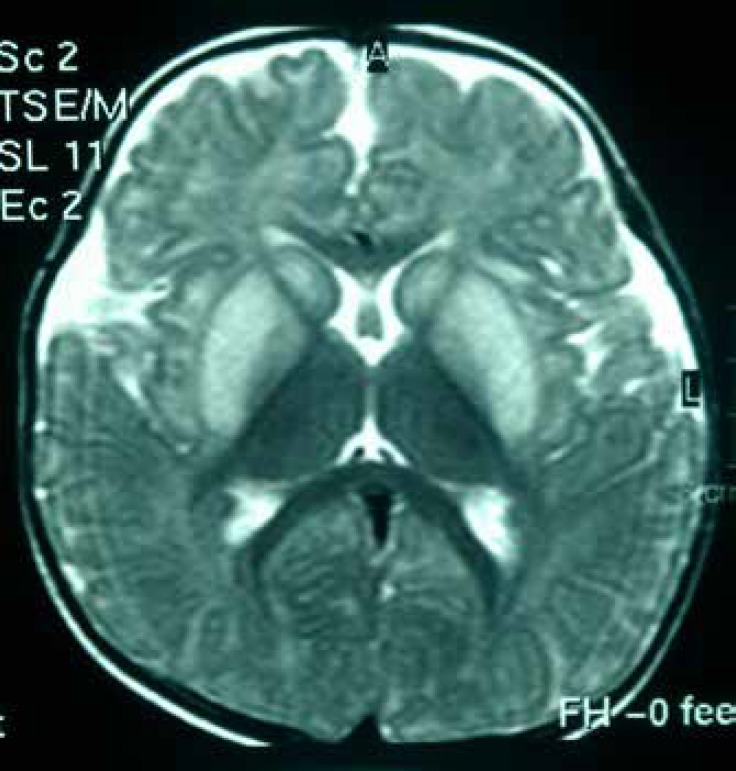
Brain MRI changes in a 4-year-old boy with propionic academia

Electroencephalography (EEG) in 4 patients was abnormal and had no special pattern. In neuroimaging data, 70% of patient had abnormal findings in brain MRI [4 patients (40%) had brain atrophy, 4 patients (40%) white matter involvement, and 3 patients (30%) globus pallidus involvement] (Figure 1).

## Discussion

Propionic acidemia is a rare metabolism disorder with recessive inheritance, caused by a defect in the activity of the mitochondrial enzyme propionyl-CoA carboxylase enzyme (7). Several hours after birth, the clinical manifestations are started with initiation of protein intake in diet. The main symptoms of this disorder are recurrent metabolic acidosis with hyperlactatemia and ketosis, which are precipitated by infection and protein intake (8).

The management of propionic acidemia is based on a low protein diet and L-carnitine to elevate propionic acid renal clearance. For acute phase of this disorder with hyperammonemia and acidosis, hydration and fluid therapy along with treatment of concomitant complications can be useful (6,9). Because of recurrent episode of metabolic acidosis despite of conservative management, the outcome of propionic acidosis is disappointing (10).

Seventy percent of our patients were offspring of consanguineous marriages. In this study, 30% of patients had a history of neonatal hospitalization because of recurrent vomiting and narcosis, and 30% had a history of neonatal hospitalization because of respiratory disorders. 

In a study by Ozand PT and collaborators on 25 patients with propionic acidemia (14 patients presented with acidosis, hyperammonemia, and thrombocytopenia in acute phase and 11 patients presented with unusual and unspecific symptoms), all the patients had a severe vomiting that they were diagnosed as intestinal obstruction in referral hospitals; Three patients had an immune disorder as the first manifestation of hospitalization; and 4 patients had an acute or chronic encephalopathy as the first manifestation of propionic acidemia. The clinical picture of propionic acidemia includes nipple and facial dysmorphia, vomiting, and hypotonia. Severe thrombocytopenia was occurred as a hallmark of the metabolic crisis.

In one patient, intracranial hemorrhage was seen, and in 3 patients, intracranial hemorrhage caused death (11). Neuropathologic findings in this disorder have not been characterized, but white matter spongiosis in neonates is seen, most prominently at the junction of gray-white matter of the temporal cortex, and also at the tracts of midbrain, pons, and medulla (12). There is a report of hemorrhage in the basal ganglia in a patient with propionic acidemia, probably due to increase in sensitivity of endothelial cells to toxic insults (13). 

Neuroimaging findings of neonates reveals white matter attenuation on computed tomography (CT) scanning and magnetic resonance imaging (MRI), which resolve after 1 year, with only a slight symmetric change in the basal ganglia (14). A study by Bergman et al. showed that the brain MRI of the patients with propionic acidemia revealed delayed myelination and cerebral atrophy (15).

In this study, in neuroimaging data, 70% of patient had abnormal findings in brain MRI, 4 (40%) had brain atrophy, 4 (40%) had white matter involvement, and 3 (30%) had globus pallidus involvement. 

In our study and 5-year follow-up, motor and cognition delay improved significantly. Fifty percent of patient with microcephaly after 6-9 month follow-up and treatment had acceptable head circumference pickup and growth weight. Fear, hallucination, and sleep disorder in one patient resolved by management therapy. Ketoacidotic attacks and loss of consciousness resolved after starting treatment. In one patient who was diagnosed at birth and treatment was started immediately, neuroimaging was normal after one year.

Seventy percent of refractory seizure was controlled with antiepileptic drug and L-carnitin. Similar to Ozand study, brain atrophy and demyelination of white matter were improved partially, but basal ganglia involvement did not resolve completely.

**In conclusion, **in our study, 70% of patients were offspring of consanguineous marriages and 60% of patients had a positive family history of neurologic disorders. So, consanguinity and positive family history are the most important factors in diagnosis of suspected patients.
